# Sex-specific alterations of cerebral blood flow in subjective tinnitus patients: an arterial spin labeling study

**DOI:** 10.3389/fnins.2026.1713913

**Published:** 2026-02-10

**Authors:** Yongli Zhang, Yu Zhao, Jixin Luan, Guolin Ma, Honglei Han

**Affiliations:** 1 Department of Otorhinolaryngology Head and Neck Surgery, China-Japan Friendship Hospital, Beijing, China; 2 Department of Radiology, China-Japan Friendship Hospital, Beijing, China; 3 Department of Radiology, Qilu Hospital of Shandong University, Jinan, Shandong, China

**Keywords:** arterial spin labeling, cerebral blood flow, magnetic resonance imaging, sex differences, tinnitus

## Abstract

**Objective:**

To explore the cerebral blood flow (CBF) alterations in chronic subjective tinnitus and to characterize the sex-related neural differences in tinnitus patients using arterial spin labeling (ASL) MRI.

**Methods:**

Fifty-two tinnitus patients and 51 healthy age- and sex-matched controls were recruited. The SPM8 was used for voxel-wise whole-brain analyses based on Gaussian random field correction. Region-of-interest (ROI) analyses were conducted according to the AAL-90 atlas. The associations between regional CBF and clinical measures, including Tinnitus Handicap Inventory (THI), Tinnitus Evaluation Questionnaire (TEQ), and Visual Analog Scale (VAS), were explored. Subgroup analyses were performed to characterize the sex-specific perfusion patterns.

**Results:**

Compared with the controls, the tinnitus patients showed relative hypoperfusion in inferior frontal gyrus, superior temporal gyrus, insula, and opercular regions; while showed relative hyperperfusion in postcentral gyrus, inferior parietal lobule, angular gyrus, supramarginal gyrus, and superior parietal lobule. The CBF in opercular inferior frontal gyrus was positively correlated with tinnitus loudness. In sex-specific analyses, compared with the female patients, the male tinnitus patients showed relative greater hyperperfusion in frontal and precentral cortices; while the female patients showed stronger increases in parietal regions. In healthy controls, males showed greater perfusion in medial prefrontal regions, and females showed stronger activity in sensorimotor and associative cortices.

**Conclusion:**

Chronic tinnitus is characterized by widespread CBF alterations in both auditory, cognitive–emotional and attentional networks. Sexually dimorphic perfusion patterns characterize distinct neuropathological mechanisms in tinnitus and support the potential of ASL perfusion imaging for precision medicine strategies in tinnitus.

## 1 Introduction

Tinnitus is a worldwide auditory health problem defined by the subjective experience of hearing sound (such as buzzing, hissing, or ringing) in the absence of an external acoustic source (Dalrymple et al., 2021; Kleinjung et al., 2024). It is a common disease in otolaryngology, with an estimated prevalence of 10–15% in the adult population and approximately 32.0% in the elderly (Chang et al., 2019; Dalrymple et al., 2021; Henry et al., 2020). Distressing tinnitus may induce depression and anxiety, as well as affect sleep. On the other hand, these factors may also aggravate tinnitus (Bhatt et al., 2017). One hypothesis proposed that similar neurobiological mechanisms may be involved in both tinnitus and depression, and the enhanced vulnerability to stress induced by phantom sounds may affect tinnitus patients more profoundly (Pinto et al., 2014). Therefore, tinnitus should be viewed as a complex neuropsychiatric disorder rather than a simple disease of the auditory system. Despite the clinical manifestations and epidemiology of tinnitus being well characterized, the neuropathophysiological mechanisms of tinnitus remain poorly understood.

To investigate the neuropathological mechanisms of tinnitus, researchers commonly use a series of neuroimaging techniques, such as functional magnetic resonance imaging (fMRI), diffusion tensor imaging (DTI) and structural MRI (sMRI) (Schmidt et al., 2018; Xu et al., 2025; Zenke et al., 2021). Among these, arterial spin labeling (ASL) is a non-invasive, tracer-free magnetic resonance perfusion imaging technique that can quantify the whole-brain cerebral blood flow (CBF) (Li X. et al., 2022; Zimmerman et al., 2021). CBF is a direct physiological index of neuronal activity because of the close relationship between the change in CBF and the metabolic demand of neural tissue. Compared with the blood oxygenation level dependent signal (BOLD) fMRI, the neuronal activity change is more sensitive and more reproducible in ASL (Chen et al., 2022). In tinnitus studies, using ASL allows us to directly investigate the altered neuronal activity in patients, and to more accurately localize and quantify the tinnitus-related neuronal abnormalities (Hu et al., 2021). Up to now, there are limited ASL studies focused on tinnitus, and few of them have systematically correlated ASL metrics with comprehensive clinical assessments or sex-based differences.

Although tinnitus affects men and women at similar rates, significant sex-based differences characterize its clinical presentation and emotional impact (Han et al., 2019). Female patients commonly report higher levels of tinnitus-related distress, anxiety, and depression, while males are more likely to describe disruptions in auditory and cognitive functions (Cederroth and Schlee, 2022; Fioretti et al., 2020). Neuroimaging studies further indicate distinct neural patterns associated with these clinical profiles: women exhibit increased activation and high-frequency oscillations in prefrontal regions—such as the orbitofrontal cortex—and limbic structures including the hippocampus and anterior cingulate cortex, which are implicated in emotional regulation and memory (Richter et al., 2021; Vanneste et al., 2012). In contrast, men show more pronounced functional changes in the primary auditory cortex and attention networks, consistent with a greater focus on the sensory aspects of tinnitus (Ruytjens et al., 2007). These findings point to sexually dimorphic neuropathological mechanisms, suggesting that tinnitus involves divergent functional reorganization patterns between sexes. Nevertheless, sex is often treated as a covariate rather than a central variable in study designs, and few neuroimaging investigations have conducted systematic subgroup analyses. Consequently, the influence of sex on tinnitus-related neural circuitry remains poorly understood.

Building on the aforementioned background, this study aimed to systematically characterize cerebral blood flow (CBF) alterations in patients with chronic subjective tinnitus using arterial spin labeling (ASL) and to compare these patterns with healthy controls. To further delineate sex-specific influences on tinnitus pathophysiology, subgroup analyses were performed stratified by sex. Clinical and ASL-MRI data were acquired from both tinnitus patients and healthy participants, followed by standardized preprocessing and advanced statistical modeling to identify whole-brain CBF alterations and their associations with clinical symptomatology. Comparative analyses between male and female tinnitus patients, as well as their respective healthy counterparts, were subsequently conducted to uncover sex-dependent neuroimaging signatures. Collectively, these findings are expected to provide mechanistic insights into the neurovascular basis of tinnitus and to establish a foundation for precision-targeted, personalized therapeutic interventions.

## 2 Materials and methods

### 2.1 Subjects

A consecutive series of 52 subjective tinnitus patients (Tinnitus, TN group) was collected from November 2020 to March 2023 in the Department of Otorhinolaryngology, China-Japan Friendship Hospital. Healthy control group (HC group, *n* = 51) were recruited from the public through social advertising and matched with subjects in TN group in terms of age, sex and education. All subjects gave informed consent before MRI examination, and the study was approved by the Ethics Committee of the China-Japan Friendship Hospital (Approval No. 2022-KY-181).

Inclusion criteria: Subjective tinnitus patients who come to the ENT department for treatment; Age range: 18–60 years; Subjective tinnitus frequency matching; Patients can independently complete tinnitus, psychological and sleep self-assessment scale.

Exclusion criteria: Objective tinnitus; Conductive hearing loss and mixed hearing loss; Organic ear disease such as otitis media or acoustic neuroma causing tinnitus; Organic brain disease such as stroke or brain tumor; Other neuropsychiatric diseases including Alzheimer’s disease, epilepsy, Parkinson’s disease and severe depression; MRI contraindication.

### 2.2 Hearing examination and tinnitus matching

All patients were tested with pure-tone audiometry and immittance audiometry in the sound-attenuated booth in the ENT department. The pure-tone average (PTA) was calculated as the mean pure-tone hearing threshold at 0.5, 1, 2, and 4 kHz to represent the level of hearing impairment. Audiometric configurations were categorized as normal, low-to-mid frequency loss, high-frequency loss, or pan-frequency loss according to hearing level. Based on the tinnitus pitch-matching results, the tinnitus frequency was divided into two groups: low-to-mid frequency (0.125–3 kHz) and high frequency (4–8 kHz).

### 2.3 Assessment of tinnitus

Clinical Characteristics, Psychological and Sleep StatusBefore the MRI scanning, all enrolled subjects filled out the Chinese versions of Tinnitus Handicap Inventory (THI), Tinnitus Evaluation Questionnaire (TEQ) and Visual Analog Scale (VAS) for tinnitus loudness in order to evaluate the psychoacoustic characteristics, emotional status and sleep quality of subjective tinnitus patients. Tinnitus Handicap Inventory (THI) was initially published in 1996 and widely used for tinnitus assessment worldwide. Tinnitus Evaluation Questionnaire (TEQ) was developed by Professor Liu Peng and convenient for measuring the psychological characteristic of tinnitus patients. Visual Analog Scale (VAS) was used for tinnitus loudness.

### 2.4 MRI data acquisition

MRI data were acquired on a 3.0T Discovery MR750 scanner (GE Healthcare, United States) using a standard head coil. Participants were scanned in supine position head first with their eyes closed and instructed to remain awake and breathe calmly and relaxed. Bilateral foam cushions were used to fix the head in place and earplugs and external earmuffs were used to reduce the noise from the scanner and protect participants’ hearing. The anterior–posterior commissure line was used as the reference for the axial scanning and the cerebral midline was used as the reference for the sagittal scanning.

(1) Conventional T2-FLAIR sequence to exclude the existence of organic lesions in the brain.

(2) 3D-high resolution sagittal T1WI sequence: slice thickness = 1.0 mm, TR = 6.7 ms, TE = 2.9 ms, FOV = 256 mm × 256 mm, matrix = 256 × 256, voxel size = 1 × 1 × 1 mm, NEX = 1, flip angle = 12°, slices = 192, acquisition time = 4 min 10 s.

(3) Single delay 3D pCASL sequence: FOV = 240 × 240 mm, slice thickness = 4.0 mm, echo time = 14.6 ms, repetition time = 4,817 ms, flip angle = 111°, slices = 36, NEX = 3, PLD = 1525 ms, scan time = 6 min 54 s.

### 2.5 CBF data processing

Whole-brain CBF maps were acquired using the GE Discovery 750MR workstation. CBF maps were converted from Dicom to NifTI format using the MRIcron plugin. Then the quality of images was checked and the images with severe deformation were eliminated. Preprocessing was done using the MATLAB-based SPM8 software with a “one-step registration method.” Finally, CBF images were registered to the Montreal Neurological Institute (MNI) standard space using the PET template in SPM8. Normalization was done using DPABI software. In this study, we chose MeanDivision as the method of normalization. The value of CBF of each voxel was divided by global mean CBF. Then the normalized images were smoothed using SPM8 with an 8 mm Gaussian smoothing kernel.

### 2.6 Structural MRI data processing

ROI-level VBM analysis was implemented by SPM12 and the CAT12.7 toolbox based on the Matlab2022a platform. The specific process were as follows: data conversion; quality checking of images; segmentation of gray matter, white matter and cerebrospinal fluid; registration of each subject’s gray matter image to the standard brain template; non-linear transformation modulation to get each subject’s GMV file; ROI segmentation of each subject’s GMV file according to the AAL brain template; Gaussian smoothing of gray matter volume files with 8 mm full-width at half-maximum Gaussian smoothing kernel; secondary quality checking of images; and total intracranial volume (TIV) calculation based on the segmented gray matter, white matter and cerebrospinal fluid volumes.

### 2.7 Statistical analysis

Statistical analysis was implemented on the basic clinical data of all participants based on the SPSS 26.0 software. The normally distributed quantitative data were described as x ± s, and independent sample *t*-test was used to compare age, education level, VAS, THI, and total intracranial volume (TIV) between the two groups. Gender comparison between the two groups was implemented based on the chi-square test. *P* < 0.05 indicated that the difference was statistically significant.

Intergroup differences in voxel-wise whole-brain zCBF values were implemented in SPM8 based on a two-sample *t*-test within the general linear model (GLM). Age, sex, education level and total intracranial volume (TIV) were implemented as the covariates. Analyses were implemented based on the whole brain using the brain-wide mask. Multiple comparisons were corrected based on the Gaussian random field (GRF) theory based on the DPABI toolbox. The voxel-wise threshold was *p* < 0.005 and the cluster-level threshold was *p* < 0.05 (two-tailed; minimum cluster size, 15 voxels).

For each subject, mean zCBF values were implemented based on clusters with significant intergroup or subgroup differences at the voxel level (defined according to AAL-90 template regions of interest (ROIs)). ROI-level group comparison was implemented based on a two-sample *t*-test. *Post hoc* multiple comparisons were corrected based on the false discovery rate (FDR) method. Statistical analyses were implemented based on SPSS (version 26.0) and applying the significance threshold *p* < 0.05 (two-tailed). The partial correlation analyses were further implemented to explore the associations between regional CBF alterations and clinical measures (VAS, THI, and TEQ) while controlling for age, sex, and education level.

## 3 Results

### 3.1 Clinical characteristics between tinnitus patients and healthy controls

There were no statistically significant differences in age, sex, years of education, TIV or the hearing thresholds between the tinnitus group and the healthy control group (all *P* > 0.05) (Tables 1.1–1.3). The tinnitus characteristics (sound quality and temporal pattern) for each patient are detailed in Table 1.4.

**TABLE 1.1:** Clinical characteristics of tinnitus patients vs. healthy controls.

Variables	HC (*n* = 51)	TN (*n* = 52)	*p*	Fisher
Sex, n (%)			0.092	2.842
Male	19 (37)	29 (56)		
Female	32 (63)	23 (44)		
Age	38 (26, 56)	37.5 (29.75, 42.25)	0.329	1474.5
Education level	16 (12, 18)	16 (15, 18)	0.426	1207.5
TIV	1448.55 ± 155.77	1487.14 ± 117.78	0.160	–1.416
VAS	/	5 (4, 6)	/	
THI	/	34 (22, 51)	/	0
TEQ	/	10 (8, 13)	/	0

**TABLE 1.2:** Right ear hearing thresholds of tinnitus patients and healthy controls.

Frequency (Hz)	HC hearing threshold (dB)	TN hearing threshold (dB)	*t*-value	*p*-value
125	11.08	12.02	0.82	0.440
250	7.45	9.23	1.45	0.196
500	6.76	7.98	1.12	0.305
1,000	8.33	9.42	0.95	0.375
2,000	8.53	9.13	0.52	0.620
4,000	10.20	10.87	0.58	0.580
8,000	12.45	12.12	–0.29	0.782
Pure-tone average	8.46	9.35	1.45	0.196

**TABLE 1.3:** Left ear hearing thresholds of tinnitus patients and healthy controls.

Frequency (Hz)	HC hearing threshold (dB)	TN hearing threshold (dB)	*t*-value	*p*-value
125	10.98	11.92	0.92	0.392
250	9.12	9.90	0.75	0.480
500	8.04	7.79	-0.24	0.818
1,000	7.75	8.27	0.50	0.635
2,000	9.80	10.77	0.93	0.387
4,000	11.76	12.16	0.38	0.716
8,000	12.94	13.53	0.57	0.588
Pure-tone average	9.34	9.71	0.48	0.646

**TABLE 1.4:** Tinnitus characteristics of tinnitus patients.

Patient	Tinnitus characteristics
1	Continuous high- pitched tinnitus
2	Continuous buzzing tinnitus
3	Continuous buzzing tinnitus
4	Continuous buzzing tinnitus
5	Continuous high- pitched tinnitus
6	Continuous high- pitched tinnitus
7	Continuous electrical humming
8	Continuous electrical humming
9	Continuous high- pitched tinnitus
10	Continuous high- pitched tinnitus
11	Continuous buzzing tinnitus
12	Continuous buzzing tinnitus
13	Intermittent hissing tinnitus
14	Continuous electrical humming
15	Continuous high- pitched tinnitus
16	Continuous high- pitched tinnitus
17	Continuous buzzing tinnitus
18	Continuous buzzing tinnitus
19	Continuous high- pitched tinnitus
20	Continuous high- pitched tinnitus
21	Continuous electrical humming
22	Continuous electrical humming
23	Continuous buzzing tinnitus
24	Continuous buzzing tinnitus
25	Intermittent hissing tinnitus
26	Intermittent buzzing tinnitus
27	Continuous high- pitched tinnitus
28	Continuous high- pitched tinnitus
29	Continuous buzzing tinnitus
30	Intermittent high- pitched tinnitus
31	Continuous hissing tinnitus
32	Continuous hissing tinnitus
33	Continuous high- pitched tinnitus
34	Continuous high- pitched tinnitus
35	Continuous high- pitched tinnitus
36	Intermittent high- pitched tinnitus
37	Continuous high- pitched tinnitus
38	Continuous buzzing tinnitus
39	Continuous buzzing tinnitus
40	Continuous buzzing tinnitus
41	Continuous high- pitched tinnitus
42	Continuous high- pitched tinnitus
43	Intermittent high- pitched tinnitus
44	Intermittent high- pitched tinnitus
45	Continuous hissing tinnitus
46	Continuous hissing tinnitus
47	Continuous buzzing tinnitus
48	Continuous buzzing tinnitus
49	Continuous high- pitched tinnitus
50	Continuous buzzing tinnitus
51	Intermittent high- pitched tinnitus
52	Continuous high- pitched tinnitus

### 3.2 Brain blood flow between tinnitus patients and healthy controls

**FIGURE 1:**
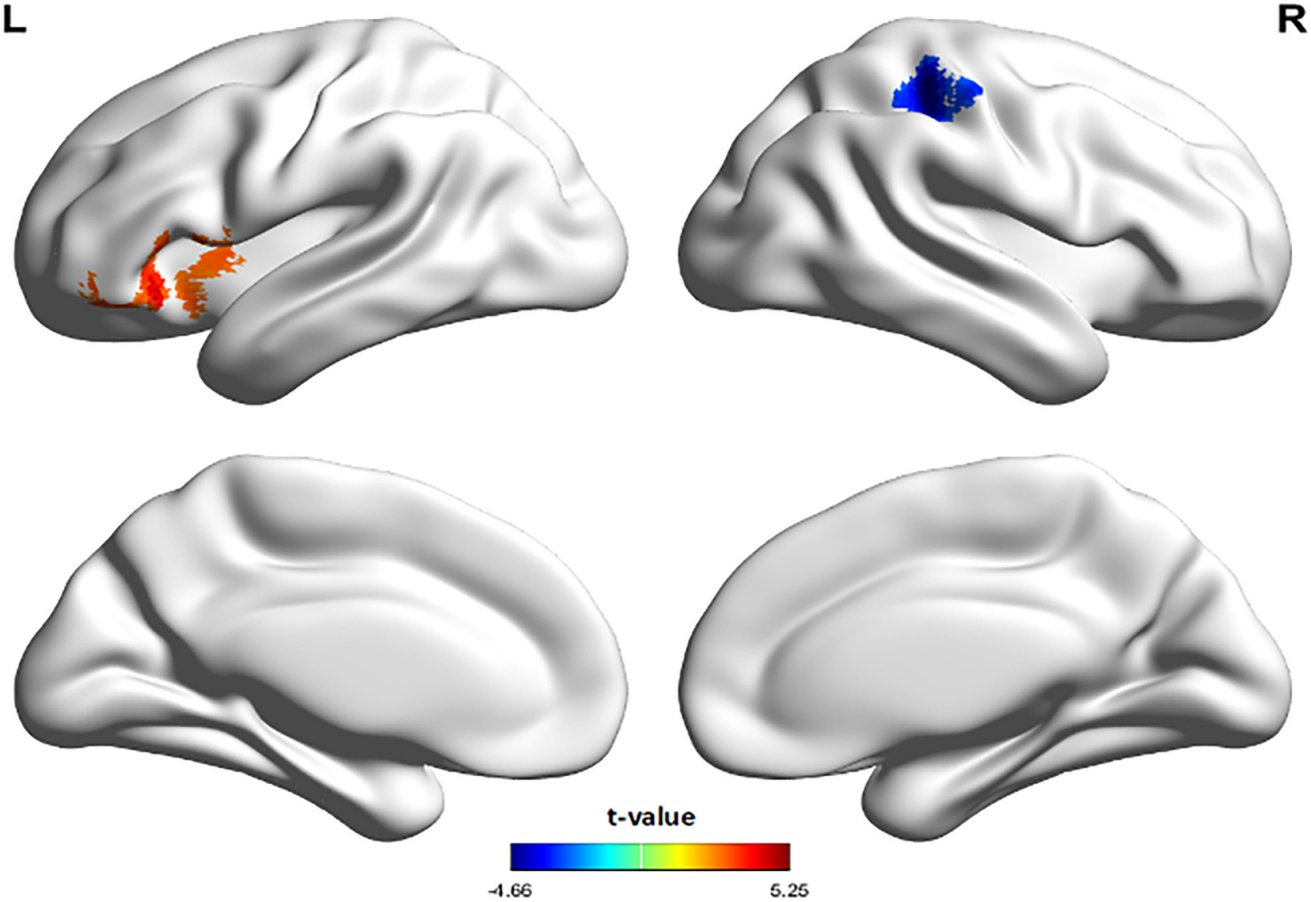
TN vs. HC zCBF group differences.

**FIGURE 2:**
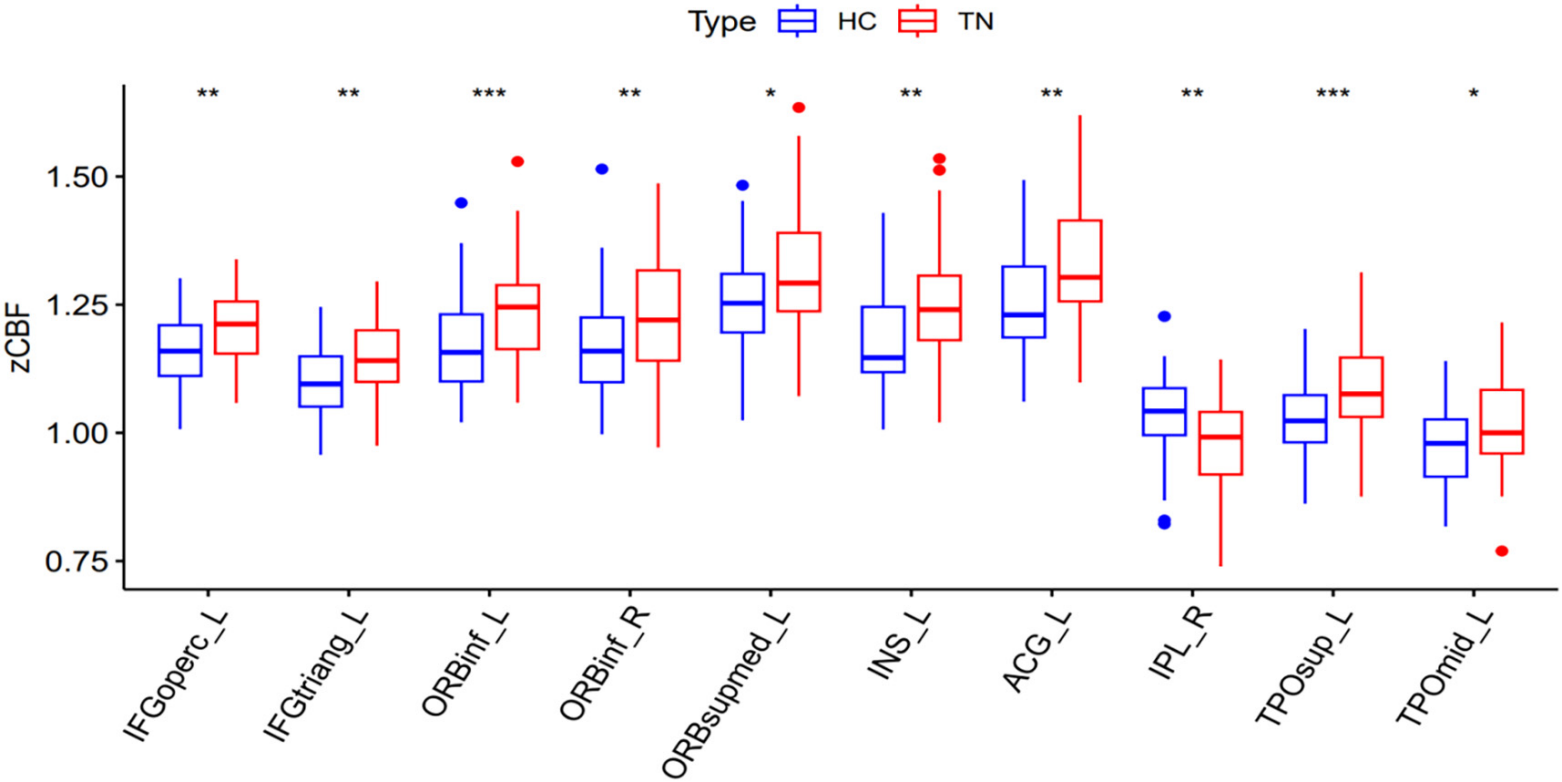
Mean zCBF group comparison between TN and HC at ROI level. zCBF, standardized cerebral blood flow; TN, Tinnitus; HC, Healthy Control; R, Right. ***p < 0.001, **p < 0.01, *p < 0.05.

The regions with zCBF differences between TN and HC groups in voxel-wise analysis are shown in Figure 1 and summarized in Table 2. Compared with HC, zCBF was significantly decreased in TN patients in orbital inferior frontal gyrus, triangular inferior frontal gyrus, superior temporal gyrus, insula, opercular inferior frontal gyrus, central operculum, and middle temporal gyrus (GRF-corrected; voxel-wise *p* < 0.005; cluster-level *p* < 0.05; cluster size ≥ 15 voxels). On the other hand, significantly increased zCBF was observed in postcentral gyrus, inferior parietal lobule, angular gyrus, supramarginal gyrus, and superior parietal lobule. The mean zCBF values of each significant cluster were shown in Figure 2 and summarized in Table 3 after the definition of the AAL template-based brain clusters.

**TABLE 2:** Significant clusters of zCBF inter-group differences at whole-brain voxel level between TN and HC.

Cluster	Cluster size (voxels)	Peak t	Cohen’s f^2^	Peak MNI coordinates
TN < HC	1,232	5.2549	0.31	–40, 26, –6
Inferior frontal gyrus, orbital	318			
Inferior frontal gyrus, triangular	279			
Temporal pole: superior temporal gyrus	228			
Insula	187			
Inferior frontal gyrus, opercular	106			
Central sulcus operculum	34			
Temporal pole: middle temporal gyrus	22			
Superior temporal gyrus	15			
TN > HC	913	–4.6648	0.24	38, –34, 50
Postcentral gyrus	422			
Inferior parietal lobule	241			
Angular gyrus	103			
Supramarginal gyrus	75			
Superior parietal lobule	51			

TN, tinnitus; HC, healthy control; zCBF, standardized cerebral blood flow. GRF corrected (voxel level *p* < 0.005, cluster level *p* < 0.05, cluster size 15), controlling for age, sex, education level, TIV as covariates.

**TABLE 3:** Inter-group comparison of mean zCBF between TN and HC.

Brain region	AAL	Mean zCBF		*t*	*p*	P.adj
		TN	HC			
Inferior frontal gyrus, opercular	IFGoperc_L	1.204 ± 0.071	1.160 ± 0.066	2.703	0.001	0.027
Inferior frontal gyrus, triangular	IFGtriang_L	1.142 ± 0.067	1.100 ± 0.066	2.768	0.002	0.027
Inferior frontal gyrus, orbital	ORBinf_L	1.236 ± 0.092	1.171 ± 0.089	3.252	0.000	0.008
Superior frontal gyrus, medial orbital	ORBsupmed_L	1.318 ± 0.121	1.258 ± 0.103	1.104	0.006	0.049
Insula	INS_L	1.246 ± 0.113	1.183 ± 0.101	2.278	0.002	0.033
Anterior cingulate gyrus	ACG_L	1.319 ± 0.123	1.254 ± 0.105	2.141	0.003	0.036
Superior temporal gyrus	TPOsup_L	1.091 ± 0.093	1.026 ± 0.070	3.287	0.000	0.002
Middle temporal gyrus	TPOmid_L	1.018 ± 0.094	0.972 ± 0.080	1.995	0.003	0.038
Inferior frontal gyrus, orbital	ORBinf_R	1.229 ± 0.110	1.171 ± 0.100	2.692	0.005	0.049
Inferior parietal lobule	IPL_R	0.983 ± 0.087	1.037 ± 0.084	–3.028	0.001	0.025

Data are displayed as mean ± standard deviation. TN, tinnitus; HC, healthy control; zCBF, standardized cerebral blood flow; Two-sample *t*-test, *p* < 0.05 (FDR corrected), controlling for age, sex, education level, TIV as covariates.

### 3.3 Association between brain blood flow differences and clinical indicators

**FIGURE 3:**
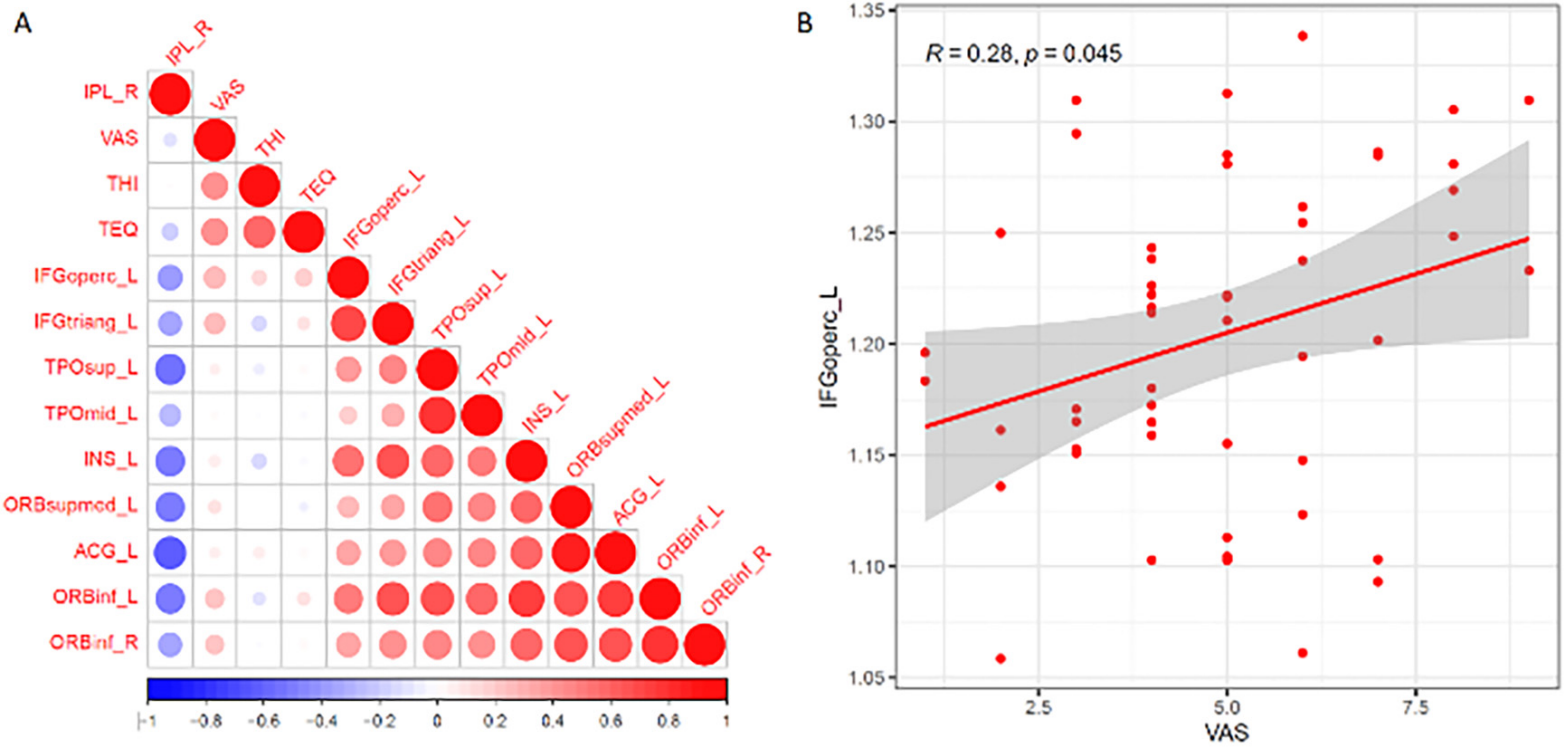
Correlation between cerebral blood flow in different brain regions and clinical scales. **(A)** Correlation matrix displaying the relationships between regional zCBF and clinical indicators (VAS, THI, and TEQ). **(B)** Scatter plot showing a significant positive correlation between zCBF in the left opercular part of the inferior frontal gyrus (IFGoperc_L) and VAS scores. The shaded area represents the 95% confidence interval.

Correlation analysis showed a significant correlation between zCBF values in the opercular inferior frontal gyrus and VAS scores in tinnitus patients (*R* = 0.28, *P* = 0.045) (Figure 3).

### 3.4 Comparison of clinical characteristics by sex in tinnitus patients

Male tinnitus patients were significantly older than female patients (median 40.52 years vs. 34.43 years, *p* = 0.025) and had a significantly larger TIV (1537.02 ± 105.23 vs. 1424.25 ± 103.20, *p* < 0.001). In terms of clinical symptoms, females had significantly higher VAS scores than males (median 5 vs. 4, *p* = 0.038), while there were no significant differences in years of education, THI, and TEQ scores between the two groups (*p* > 0.05) (Table 4).

**TABLE 4:** Comparison of clinical data between male and female tinnitus patients.

Variables	Male (*n* = 29)	Female (*n* = 23)	*p*-value	Fisher
Age, median (Q1, Q3)	40.52 (33, 48)	34.43 (28, 40)	0.025*	2.317
Edu, median (Q1, Q3)	16 (12, 18)	16 (16, 18)	0.439	
TIV, mean ± SD	1537.02 ± 105.23	1424.25 ± 103.20	< 0.001**	3.88
VAS, median (Q1, Q3)	4 (3, 5)	5 (4, 6)	0.038*	
THI, median (Q1, Q3)	30 (18, 48)	38 (25, 54)	0.220	266.5
TEQ, median (Q1, Q3)	11 (8, 14)	10 (8, 12)	0.373	0.899

*p < 0.0 5,

**p < 0.01.

### 3.5 Brain blood flow changes by sex in tinnitus patients

**FIGURE 4:**
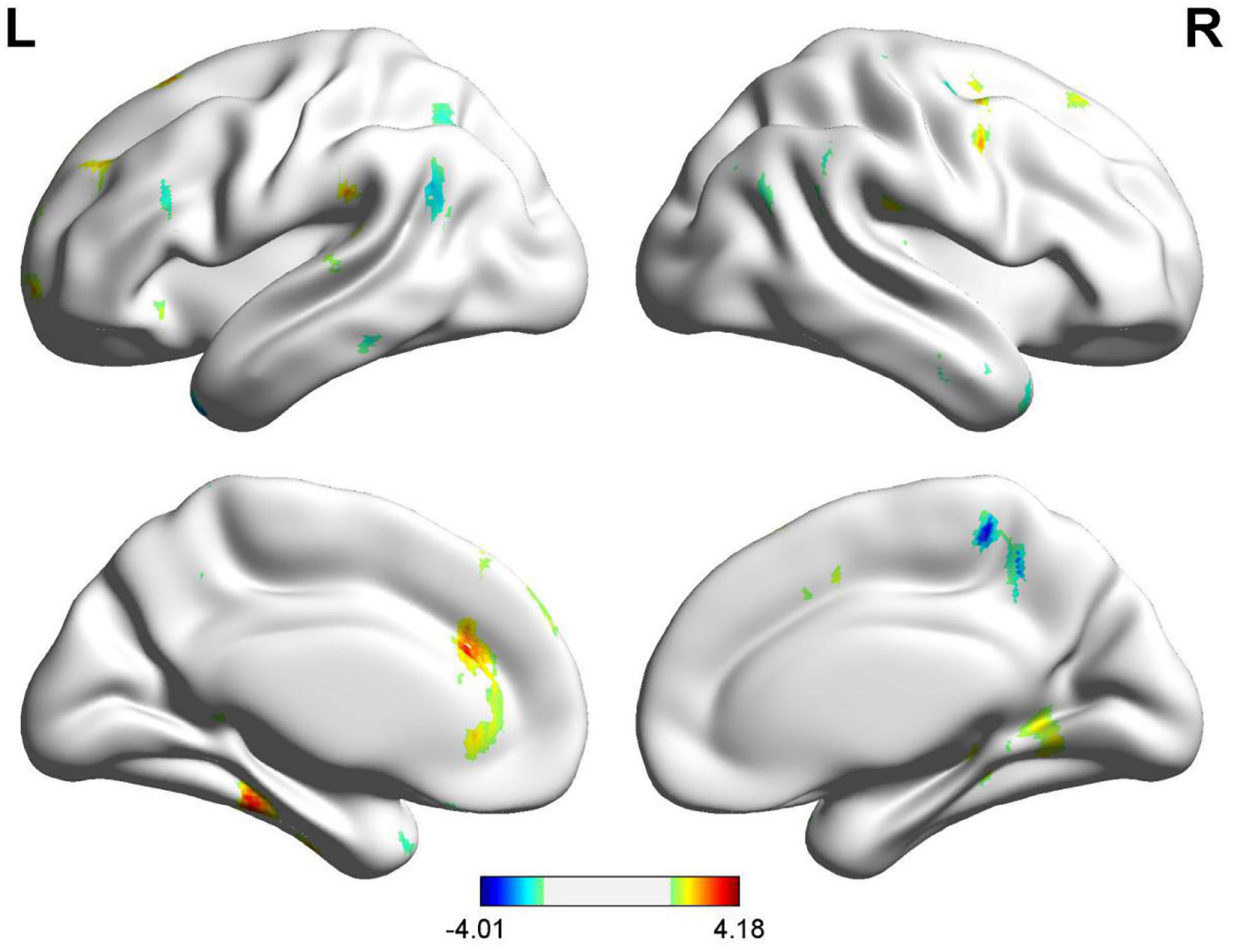
Male vs. female zCBF group differences.

**FIGURE 5:**
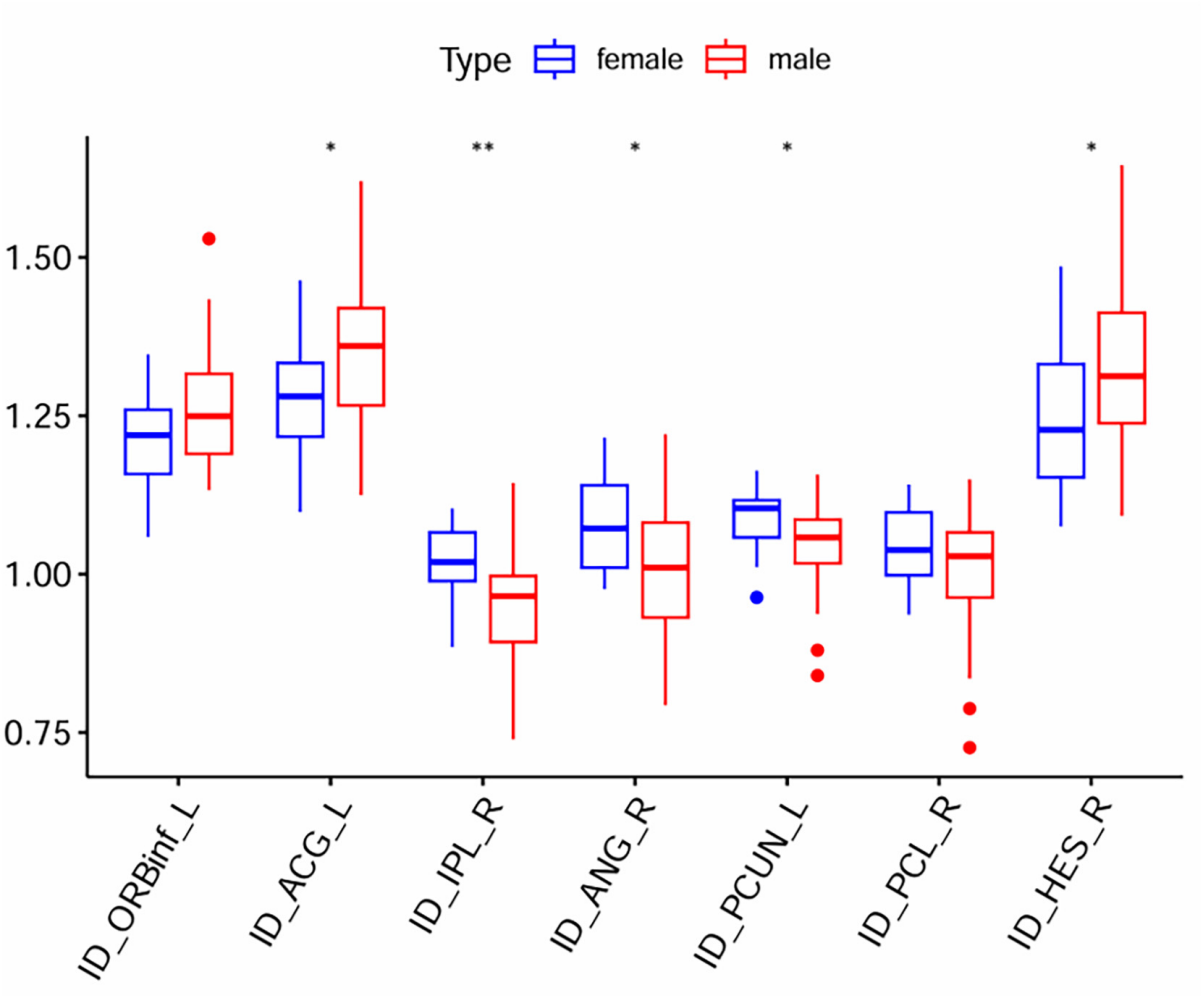
Mean zCBF group comparison between male and female at ROI level. zCBF, standardized cerebral blood flow; R, Right. **p < 0.01, *p < 0.05.

In voxel-wise whole-brain analysis, sex-related differences of zCBF in tinnitus patients are shown in Figure 4 and summarized in Table 5. Compared with females, male patients revealed significantly increased zCBF in frontal lobe, middle frontal gyrus, right cerebrum, and precentral gyrus (GRF-corrected; voxel-wise *p* < 0.005; cluster-level *p* < 0.05; cluster size ≥ 15 voxels). Furthermore, male patients demonstrated significantly decreased zCBF in right cerebrum, precentral gyrus, frontal lobe, and paracentral lobule. ROI-level intergroup differences in mean zCBF values in each significantly activated cluster according to the AAL template are shown in Figure 5 and Table 6.

**TABLE 5:** Significant clusters of zCBF inter-group differences at whole-brain voxel level between male and female tinnitus patients.

Cluster	Cluster size (voxels)	Peak t	Cohen’s f^2^	Peak MNI coordinates
Male > female	30	4.183	0.33	36, 0, 46
Frontal lobe	30			
Middle frontal gyrus	30			
Right cerebrum	30			
Precentral_R (aal)	25			
White matter	21			
Male < female	32	–3.609	0.25	6, –46, 46
Right cerebrum	32			
Precuneus_R (aal)	25			
White matter	20			
Frontal lobe	19			
Paracentral Lobule	19			

CBF, standardized cerebral blood flow. GRF corrected (voxel level *p* < 0.005, cluster level *p* < 0.05, cluster size 15), controlling for age, education level, TIV as covariates.

**TABLE 6:** Inter-group comparison of mean zCBF between male and female tinnitus patients.

Brain region	AAL	Mean zCBF		*T*-value	*P*	P.adj
		Male	Female			
Left anterior cingulate gyrus	ID_ACG_L	1.352 ± 0.131	1.278 ± 0.100	–1.079	0.025	0.025
Right angular gyrus	ID_ANG_R	1.008 ± 0.106	1.079 ± 0.077	1.330	0.008	0.008
Right Heschl’s gyrus	ID_HES_R	1.330 ± 0.140	1.247 ± 0.118	–1.145	0.025	0.025
Right inferior parietal lobule	ID_IPL_R	0.956 ± 0.096	1.017 ± 0.060	1.142	0.008	0.008
Left inferior frontal gyrus, orbital	ID_ORBinf_L	1.260 ± 0.096	1.206 ± 0.078	–1.091	0.030	0.030
Right paracentral lobule	ID_PCL_R	0.995 ± 0.101	1.044 ± 0.061	1.777	0.033	0.033
Left precuneus	ID_PCUN_L	1.039 ± 0.075	1.084 ± 0.047	1.166	0.012	0.012

Data are displayed as mean ± standard deviation. TN, tinnitus; HC, healthy control; zCBF, standardized cerebral blood flow; Two-sample *t*-test, *p* < 0.05 (FDR corrected), controlling for age, sex, education level, TIV as covariates.

### 3.6 Correlation between CBF and clinical indicators (sex-specific)

**FIGURE 6:**
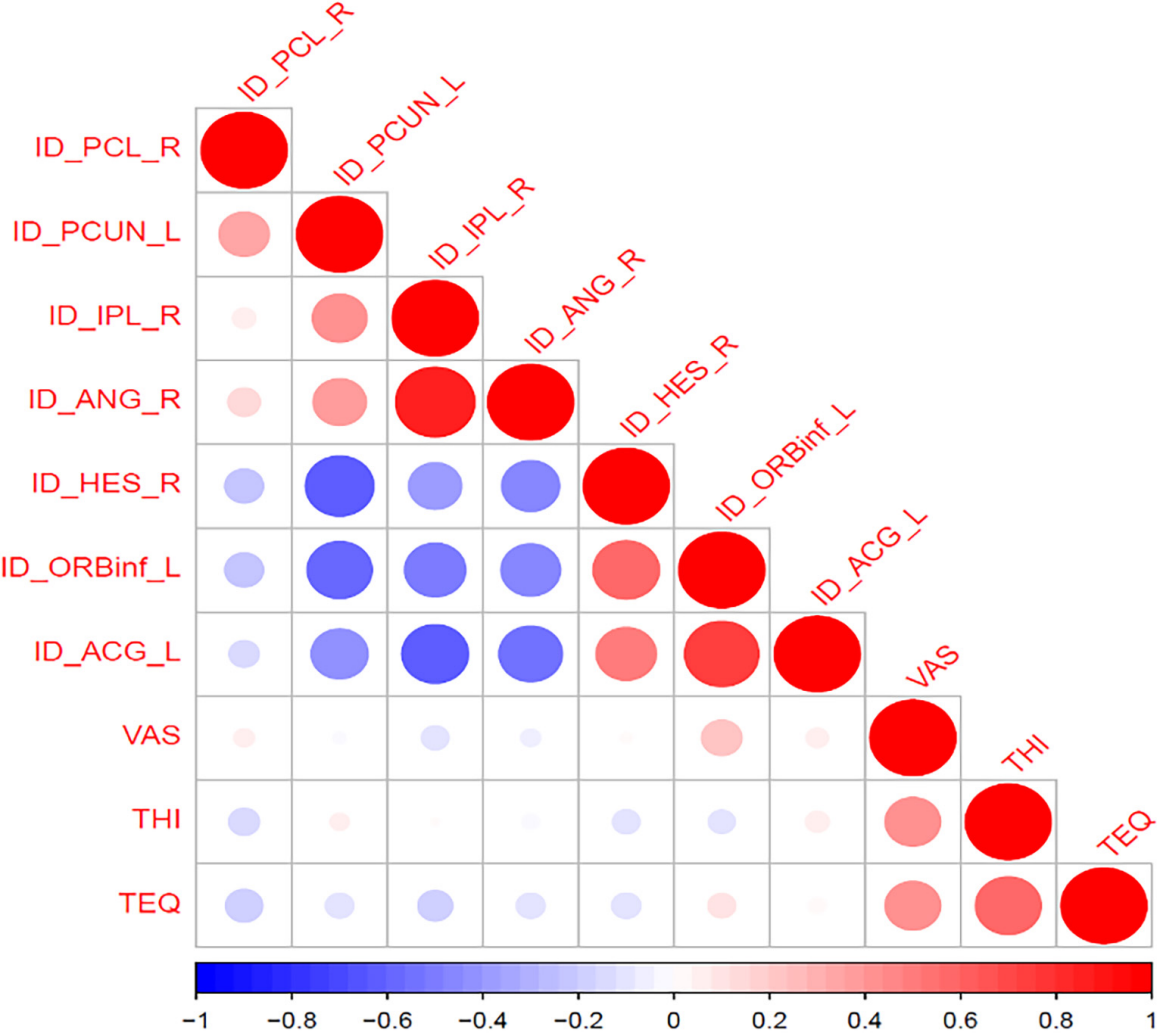
Correlation between CBF in differential brain regions and clinical scales in male and female tinnitus patients.

No significant brain regions were found in the correlation analysis (Figure 6).

### 3.7 Comparison of clinical characteristics by sex in healthy controls

Analysis of the study data showed that male healthy controls had significantly larger intracranial volume than females (1573.14 vs. 1367.72, *p* < 0.001) and longer years of education (16 vs. 12 years, *p* = 0.003), but no significant age difference (*p* = 0.083) (Table 7).

**TABLE 7:** Comparison of clinical data between male and female healthy controls.

Variables	Male (*n* = 20)	Female (*n* = 30)	Statistic	*p*-value
Age, median (Q1, Q3)	33.5 (27.75, 55.25)	54.5 (26.25, 61)	*z* = –1.732	0.083
Edu, median (Q1, Q3)	16 (12, 19)	12 (9, 16)	*z* = –2.969	0.003**
TIV, mean ± SD	1573.14 ± 158.31	1367.72 ± 114.33	*t* = 5.437	<0.001***

**p < 0.01,

***p < 0.001.

### 3.8 Brain blood flow changes by sex in healthy controls

**FIGURE 7:**
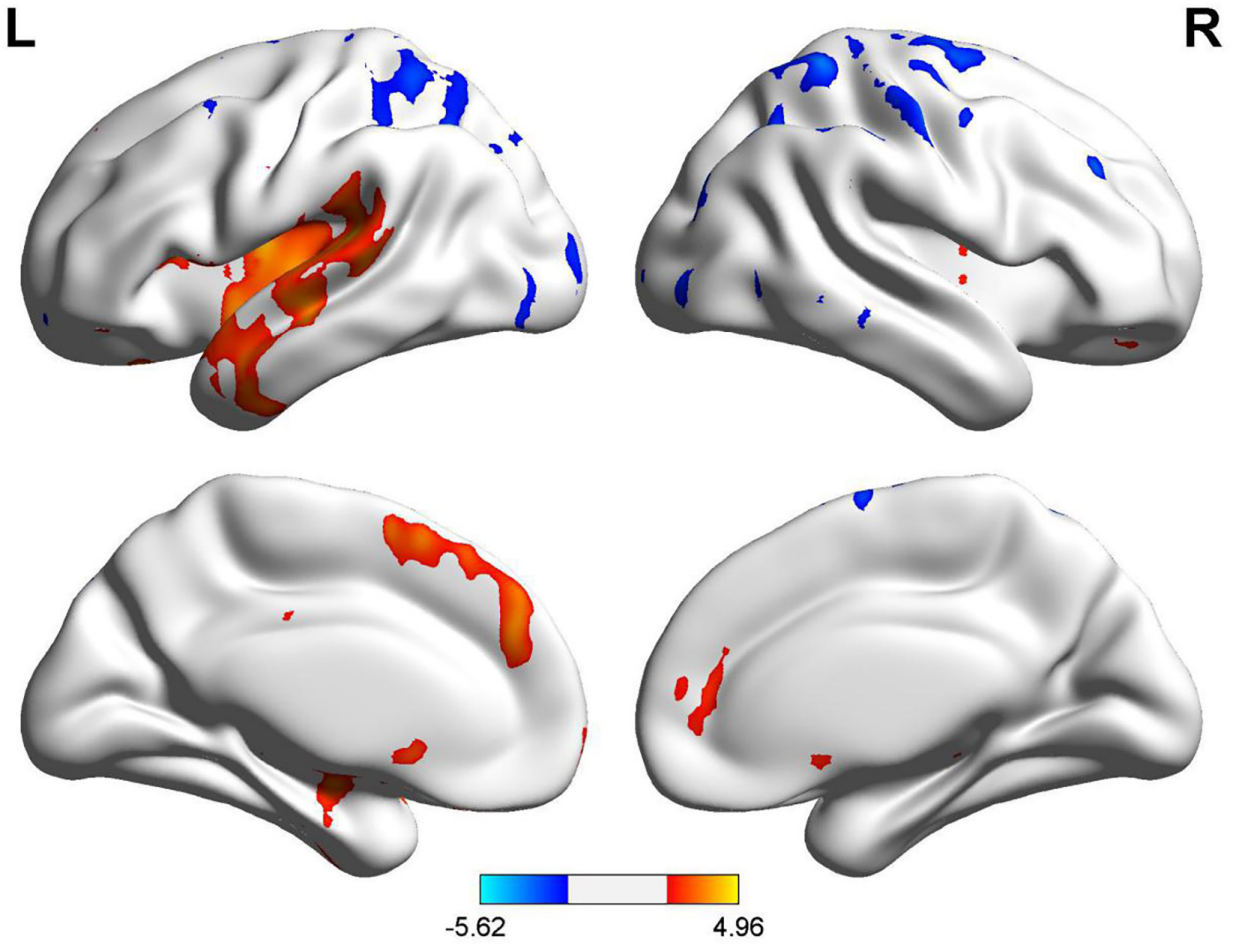
Male vs. female zCBF group differences.

**FIGURE 8:**
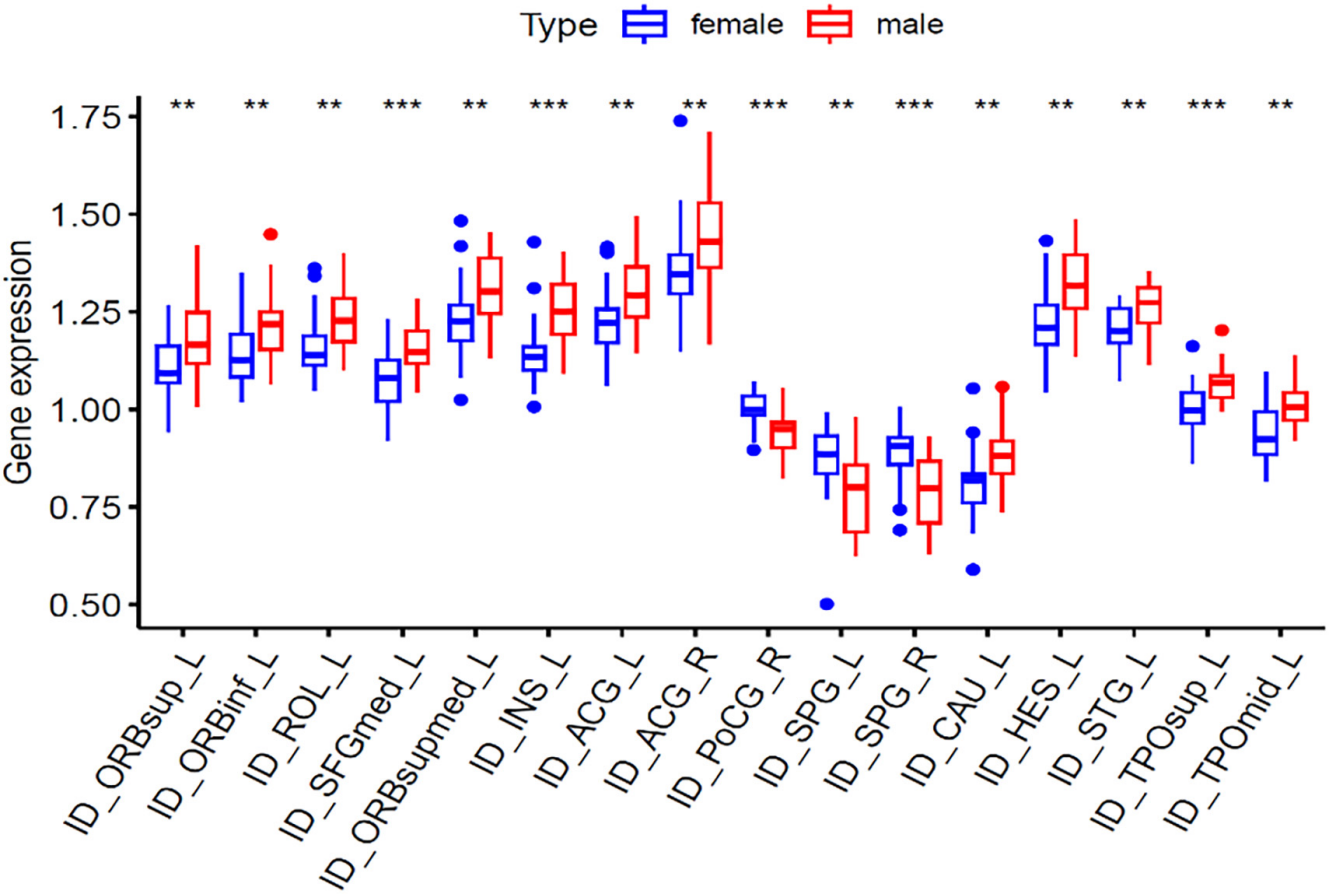
Mean zCBF group comparison between male and female at ROI level. zCBF, standardized cerebral blood flow; TN, Tinnitus; HC, Healthy Control; R, Right. ***p < 0.001, **p < 0.01.

In voxel-wise whole brain analysis, sex-related zCBF differences in healthy controls were displayed in Figure 7 and Table 8. Compared with females, males presented higher zCBF in prefrontal region, i.e., left medial superior frontal gyrus (Frontal_Sup_Medial_L), left supplementary motor area (Supp_Motor_Area_L), and left superior frontal gyrus (Frontal_Sup_L). Conversely, males presented lower zCBF in sensorimotor cortices, i.e., right postcentral gyrus (Postcentral_R), right superior parietal lobule (Parietal_Sup_R), and right precentral gyrus (Precentral_R), as well as in posterior associative areas, i.e., right superior frontal gyrus (Frontal_Sup_R), right inferior parietal lobule (Parietal_Inf_R), right middle frontal gyrus (Frontal_Mid_R), right angular gyrus (Angular_R), and right supramarginal gyrus (SupraMarginal_R). All results survived GRF correction (voxel-level *p* < 0.005, cluster-level *p* < 0.05, cluster size ≥ 15 voxels). The mean zCBF based on AAL template in ROI level are displayed in Figure 8 and Table 9.

**TABLE 8:** Significant clusters of zCBF inter-group differences at whole-brain voxel level between male and female healthy controls.

Cluster	Cluster size (voxels)	Peak t	Peak MNI coordinates
Male > female	458	4.9567	–2, 44, 40
Frontal_Sup_Medial_L	337		
Supp_Motor_Area_L	70		
Frontal_Sup_L	44		
Male < Female	2075	–5.6238	50, –14, 58
Postcentral_R	452		
Parietal_Sup_R	430		
Precentral_R	339		
Frontal_Sup_R	282		
Parietal_Inf_R	205		
Frontal_Mid_R	130		
Angular_R	111		
SupraMarginal_R	44		

CBF, standardized cerebral blood flow. GRF corrected (voxel level *p* < 0.005, cluster level *p* < 0.05, cluster size 15), controlling for age, education level, TIV as covariates.

**TABLE 9:** Inter-group comparison of mean zCBF between male and female healthy controls.

Brain region	AAL	Mean zCBF		*t*	*P*	P.adj
		Male	Female			
Left anterior cingulate gyrus	ID_ACG_L	1.312 ± 0.101	1.220 ± 0.093	–1.861	0.002	0.015
Right anterior cingulate gyrus	ID_ACG_R	1.453 ± 0.136	1.353 ± 0.117	–1.278	0.006	0.039
Left caudate nucleus	ID_CAU_L	0.882 ± 0.086	0.810 ± 0.089	–2.884	0.006	0.038
Left Heschl’s gyrus	ID_HES_L	1.325 ± 0.099	1.225 ± 0.088	–2.939	0.000	0.002
Left insula	ID_INS_L	1.254 ± 0.090	1.141 ± 0.082	–2.138	0.005	0.034
Left inferior frontal gyrus, orbital	ID_ORBinf_L	1.214 ± 0.092	1.145 ± 0.078	–3.134	0.000	0.001
Left superior frontal gyrus, orbital	ID_ORBsup_L	1.186 ± 0.105	1.104 ± 0.072	–2.079	0.002	0.015
Left superior frontal gyrus, medial orbital	ID_ORBsupmed_L	1.311 ± 0.094	1.226 ± 0.096	–1.851	0.008	0.046
Right postcentral gyrus	ID_PoCG_R	0.931 ± 0.058	1.002 ± 0.043	3.953	0.000	0.001
Left rolando operculum	ID_ROL_L	1.230 ± 0.084	1.167 ± 0.072	2.851	0.001	0.010
Left superior frontal gyrus, medial	ID_SFGmed_L	1.160 ± 0.069	1.075 ± 0.070	3.115	0.000	0.004
Left superior parietal lobule	ID_SPG_L	0.779 ± 0.108	0.874 ± 0.092	–2.986	0.005	0.035
Right superior parietal lobule	ID_SPG_R	0.788 ± 0.094	0.881 ± 0.073	–3.432	0.001	0.007
Left superior temporal gyrus	ID_STG_L	1.266 ± 0.063	1.205 ± 0.057	–4.033	0.001	0.007
Left middle temporo-parietal-occipital assoc. cortex	ID_TPOmid_L	1.017 ± 0.060	0.945 ± 0.079	–1.946	0.000	0.001
Left superior temporo-parietal-occipital assoc. cortex	ID_TPOsup_L	1.068 ± 0.053	1.001 ± 0.067	-1.156	0.000	0.002

Data are displayed as mean ± standard deviation. zCBF, standardized cerebral blood flow; Two-sample *t*-test, *p* < 0.05 (FDR corrected), controlling for age, education level, TIV as covariates.

### 3.9 Comparison of clinical characteristics between female tinnitus patients and female healthy controls

There were no statistically significant differences in age, education level, or TIV between the female tinnitus patients group and female healthy controls group (all *P* > 0.05) (Table 10).

**TABLE 10:** Comparison of clinical data between female tinnitus patients and female healthy controls.

Variables	HC (*n* = 32)	TN (*n* = 23)	*p*	Fisher
Age, median (Q1, Q3)	50.5 (26, 60)	34 (27, 38.5)	0.061	258
Edu, median (Q1, Q3)	13.90 ± 4.32	15.30 ± 3.62	0.079	
TIV, mean ± SD	1377.59 ± 111.41	1424.25 ± 103.2	0.116	1.599
VAS, median (Q1,Q3)	0 (0, 0)	4 (3, 6)	/	
THI, median (Q1, Q3)	0 (0, 0)	38 (25, 54)	/	736
TEQ, median (Q1, Q3)	0 (0, 0)	10 (7.5, 13)	/	736

### 3.10 Brain blood flow changes in female tinnitus patients

**FIGURE 9:**
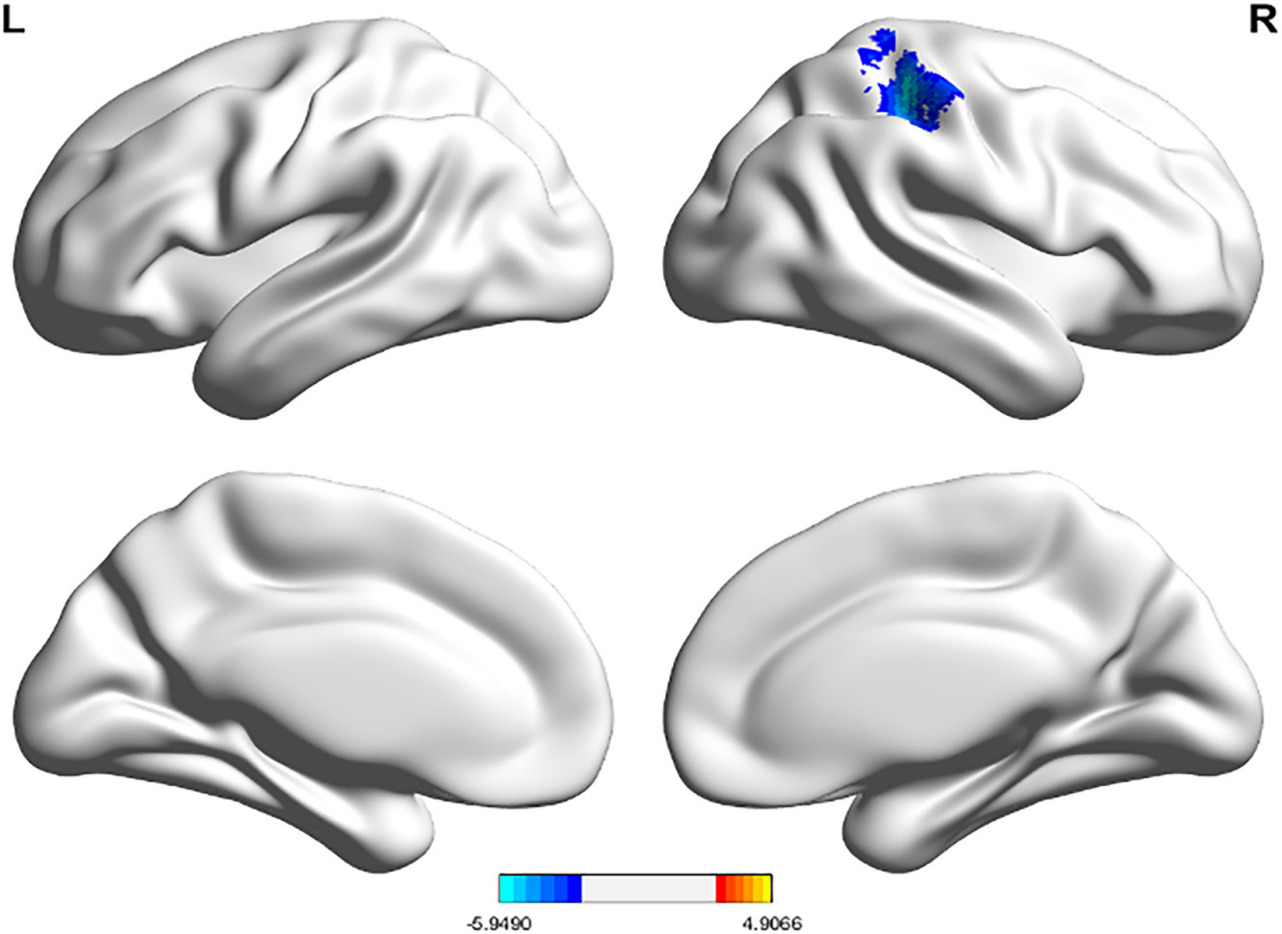
Female tinnitus patients vs. female healthy controls zCBF group differences.

**FIGURE 10:**
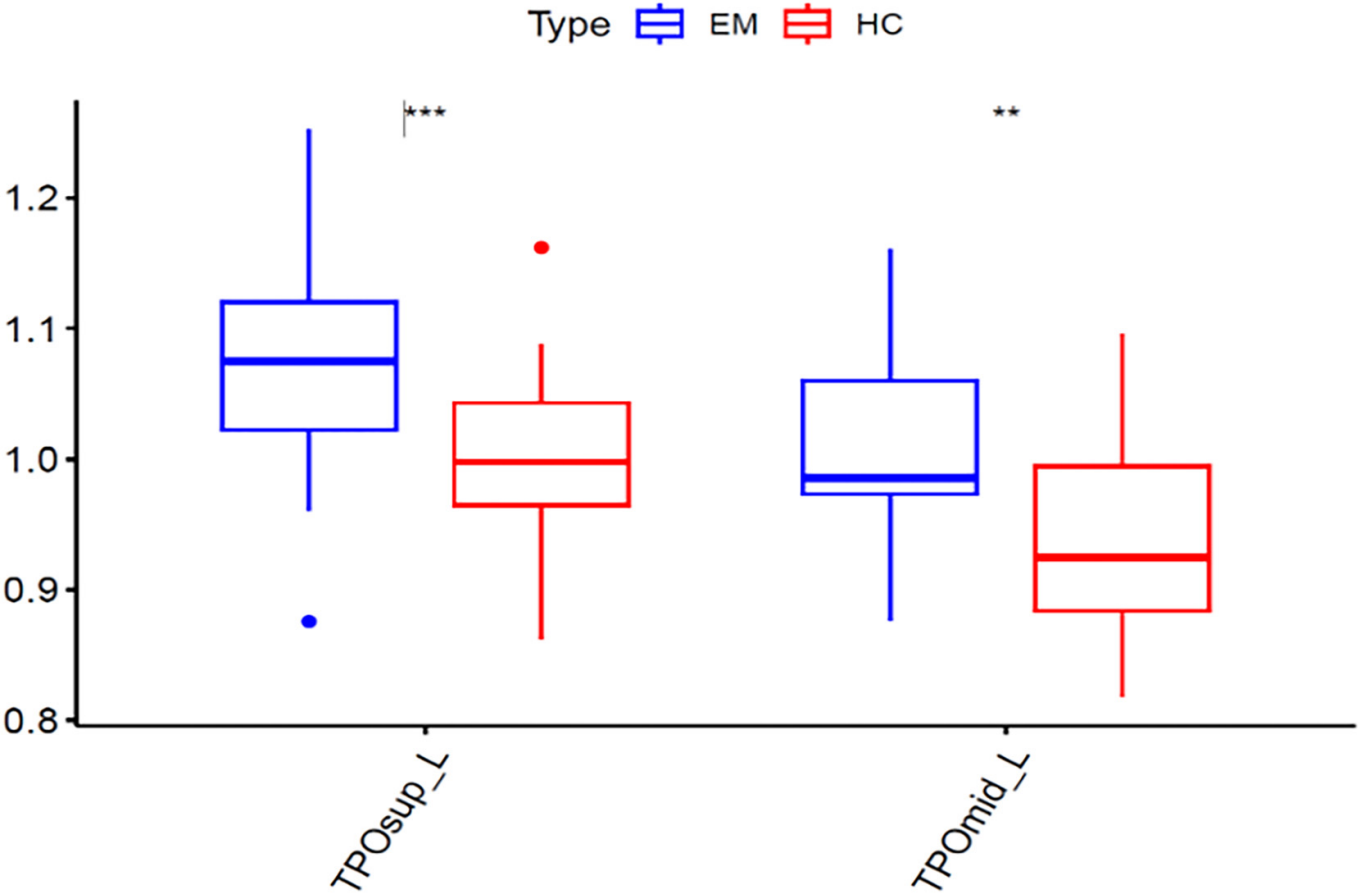
Mean zCBF group comparison between female tinnitus patients and female healthy controls at ROI level. zCBF, standardized cerebral blood flow; R, Right. ***p < 0.001, **p < 0.01.

A voxel-based whole-brain analysis of zCBF differences between female tinnitus patients and female healthy controls is shown in Figure 9 and Table 11. Compared to female healthy controls, female tinnitus patients showed significantly increased zCBF in the postcentral gyrus. This difference was corrected using GRF (voxel level *p* < 0.005, cluster level *p* < 0.05, cluster size 15). The differences in zCBF for each significant cluster at the AAL template’s ROI level are shown in Figure 10 and Table 12.

**TABLE 11:** Comparison of clinical data between female tinnitus patients and female healthy controls.

Cluster	Cluster size (voxels)	Peak t	Peak MNI coordinates
TN > HC	30	–5.949	42, –34, 54
Parietal lobe	1239		
Postcentral_R (aal)	684		
Postcentral gyrus	646		
White matter	597		
Gray matter	583		
Inferior parietal lobule	384		
Parietal_Inf_R (aal)	310		
brodmann area 40	297		
Parietal_Sup_R (aal)	207		
Sub-Gyral	171		
SupraMarginal_R (aal)	142		

CBF, standardized cerebral blood flow. GRF corrected (voxel level *p* < 0.005, cluster level *p* < 0.05, cluster size 15), controlling for age, education level, TIV as covariates.

**TABLE 12:** Comparison of mean zCBF between female tinnitus patients and female healthy controls.

Brain Region	AAL	Mean zCBF		*p*	*P.adj*	*t*
		EM	HC			
Temporal_Pole_Mid_L	ID_TPOmid_L	1.016 ± 0.079	0.945 ± 0.079	0.022	0.002	–1.079
Temporal_Pole_Sup_L	ID_TPOsup_L	1.076 ± 0.087	1.001 ± 0.067	0.010	0.001	–1.029

## 4 Discussion

In this study, we employed ASL imaging to explore the possible modifications of brain blood flow patterns in tinnitus patients with respect to healthy controls and extended this investigation to the examination of sex-related differences of brain function in both groups of subjects. Our results shed light on the neural mechanisms involved in tinnitus and strongly support the recommendation that sex is a critical biological variable in neuroimaging research.

In accordance with ASL-derived zCBF results, compared with the healthy controls, the subjective tinnitus patients showed significantly reduced perfusion in inferior frontal gyrus, temporal lobe and insula—regions that are components of fronto-temporal-limbic network—while increased perfusion in parietal cortices involved in sensory and attentional processing. The spatial distribution pattern suggests that tinnitus may not be localized in auditory cortex, but may involve large-scale reorganization within emotion- and cognition-related control network as well as sensory-attention system (Rauschecker et al., 2015; Zimmerman et al., 2021).

Remarkably, regions with reduced CBF, including orbitofrontal gyrus and insula, are highly conserved core nodes of emotional and cognitive control network. The orbitofrontal cortex is involved in emotional regulation, reward–punishment evaluation, and behavioral decision-making. Hypoperfusion in orbitofrontal cortex may reflect defects of emotional processing and impairments of regulation of negative affect in tinnitus patients (Rauschecker et al., 2015). Insula is the core region with principal hubs of multisensory and interoceptive integration that showed hypoperfusion that may represent defects of auditory–emotional coupling in the pathophysiology of tinnitus (Xu et al., 2019). In addition, hypoperfusion in superior temporal gyrus may represent either a compensation for the persistent input from chronically tinnitus-affected brain areas or reorganization of auditory cortex. In line with this notion, previous ASL and fMRI reports have shown perfusion and metabolic abnormalities in secondary auditory cortices and temporal pole in chronic tinnitus, which may mediate either persistence or perceptual characteristics of this disorder (Jaroszynski et al., 2022; Wineland et al., 2012). These decreases in perfusion may represent enhanced local inhibitory activity, decreased neural metabolism, or a stable regulatory state resulting from prolonged neuroplastic changes (Hu et al., 2021). In contrast, regions with increased CBF, including the postcentral gyrus and parietal cortices, were predominantly involved in sensory processing and attentional control. The postcentral gyrus, which represents the primary somatosensory cortex, demonstrated hyperperfusion that may reflect somatosensory modulation of tinnitus, with bodily sensations (e.g., cervical or temporomandibular muscle tension) modulating tinnitus loudness or pitch (Sanchez et al., 2007). The parietal cortex, comprising the inferior parietal lobule, angular gyrus, and supramarginal gyrus, is critically involved in multisensory processing and directing attentional resources to either process persistent tinnitus-related signals or suppress them, reflecting the attentional and cognitive features commonly associated with tinnitus patients (Jaroszynski et al., 2022; Salvari et al., 2023; Wineland et al., 2012). Overall, these results provide convergent evidence that tinnitus should not be viewed as an isolated auditory cortical abnormality, but instead as a disorder manifesting a functional reorganization of neural activity within distributed auditory, emotional, attentional, and sensorimotor networks.

This study further explored the relationship between CBF alterations and clinical features of tinnitus. We found that there was a significantly positive correlation between zCBF values in the left opercular part of inferior frontal gyrus (opercular IFG) and tinnitus loudness measured by visual analog scale (VAS) (*R* = 0.28, *P* = 0.045). Since VAS is widely accepted as subjective measure of tinnitus loudness, our results further highlighted the potential clinical implication of perfusion abnormalities within prefrontal circuitry. The orbitofrontal and prefrontal cortices are highly integrative stations at the crossroads of auditory, affective, and cognitive processing, and are thought to play a central role in higher-order appraisal and evaluative processes of stimuli and perception (Araneda et al., 2018). Previous studies have provided supportive evidences for the involvement of prefrontal activity in tinnitus: Kim et al. reported that prefrontal activity, including orbitofrontal subdivisions, predicts short-term improvement in tinnitus loudness and distress (Kim et al., 2016). Seydell-Greenwald et al. found significant correlations between ventral prefrontal BOLD activity and tinnitus loudness (Seydell-Greenwald et al., 2012). De Ridder et al. proposed an integrative model in which prefrontal areas exert top-down modulation on auditory–emotional interactions that give rise to the subjective perception of tinnitus (De Ridder et al., 2014). Under this hypothesis, the positive correlation between CBF in the left opercular IFG and VAS scores observed in the present study may reflect increased neuro-metabolic demand due to the elevated appraisal of tinnitus loudness and tinnitus-related distress, or compensate for the engagement of top-down cognitive regulatory processes in face of persistent auditory stimuli intrusions. Meanwhile, recent findings by Araneda et al. are also consistent with our results: they also reported significant positive correlation between prefrontal activity and tinnitus loudness, and suggested that perfusion changes in prefrontal cortex may represent a putative neural biomarker involved in the process of tinnitus loudness perception (Araneda et al., 2018). Furthermore, their results also provided mechanistic support for prefrontal cortex as a promising target for neuromodulatory interventions. Transcranial magnetic stimulation or transcranial direct current stimulation) for tinnitus, aimed at restoring network homeostasis and reducing tinnitus symptomatology (Elyssa Kok et al., 2021).

On examination of female patients, we found significantly increased CBF in right postcentral gyrus. Postcentral gyrus is an essential part of primary somatosensory cortex, and the hyperperfusion may be due to the abnormal activity of somatosensory–auditory pathway integration. Leaver et al. also found increased functional connectivity between somatosensory and auditory cortices in tinnitus patients and the connectivity was positively correlated to tinnitus-related distress (Leaver et al., 2011). It has been reported that female patients may report more tinnitus-related distress and higher anxiety level in general. The increased anxiety level may be due to the abnormal activity of somatosensory–auditory pathway integration and meanwhile, the high anxiety level may aggravate the negative perceptual experience of tinnitus.

In addition, we also found significantly increased CBF in middle and superior temporal pole of female patients. These two poles are critical stations in auditory and limbic networks and are responsible for emotional regulation and auditory information integration. The increased CBF in these two poles may be due to the differential activity of auditory and limbic networks and/or the affective coping strategy in persistent auditory stimulation. Chen et al. proposed that tinnitus, hyperacusis, and phonophobia may be due to the abnormal interaction in auditory–limbic–arousal–cerebellar network (Chen et al., 2015). Evolutionarily, women are more sensitive to their auditory environment and are more likely to be aware of and process tinnitus signals. In agreement with this, Balaresque et al. (2025) also found that women’s cochlear sensitivity is on average 2 dB better than that of men and potentially have stronger central auditory pathway function (Balaresque et al., 2025; Li W. et al., 2022). These biological differences in auditory processing may contribute to the stronger abnormalities of somatosensory–auditory pathway in female tinnitus patients.

In comparison, we found that male patients had increased perfusion in frontal lobe and precentral gyrus which are responsible for cognitive control and motor planning. We supposed that this increased perfusion was due to the compensatory recruitment of frontal networks to suppress tinnitus-related signals. Increased activity in prefrontal cortex has been reported to be associated with adaptive strategies in tinnitus management (Becker et al., 2022). Therefore, men may prefer to rely on cognitive control mechanism to regulate tinnitus perception and we found that there was more hyperperfusions in prefrontal regions. Interestingly, male patients also showed significant hypoperfusion in precuneus and parietal cortex. Precuneus is a core hub in DMN and is responsible for self-referential processing and episodic memory. The dysfunction in these two regions may contribute to the abnormalities in self-awareness and memory as well as spatial navigation. Schmidt et al. (2013) found tinnitus-related alterations in DMN activity (Schmidt et al., 2013) and our results further demonstrated that male patients may have more obvious disruption in specific DMN subsystems.

These sex-specific perfusion patterns may represent different neural strategies to deal with tinnitus. For female patients, neuromodulatory strategies targeting abnormal somatosensory–auditory integration by TMS or transcranial direct current stimulation (tDCS) directed at the right postcentral gyrus and temporal–parietal regions may provide potential therapeutic opportunities (De Ridder et al., 2011). For male patients, similar strategies targeting prefrontal–parietal network balance restoration in combination with cognitive rehabilitation to enhance DMN functional integration may be more beneficial for reducing symptoms (Wiegand et al., 2019).

Although the present study advanced much, several limitations remain. First, our sample size was relatively small, especially for subgroup analyses, which may have reduced statistical power and resulted in nonsignificant correlations. Second, because of the cross-sectional design, we could not determine causal relationships between CBF alterations and the tinnitus course. Future longitudinal studies are needed to clarify dynamic perfusion changes across the entire course of tinnitus onset and progression. Third, we adjusted for age, education, and total intracranial volume (TIV); however, it cannot be completely ruled out that other unmeasured confounders, such as the severity of hearing loss, tinnitus duration, or even some latent psychological variables, affected our results. In the future, more refined stratification strategies and multimodal brain mapping approaches, such as resting-state fMRI and diffusion tensor imaging, should be employed to achieve a more comprehensive understanding of both the functional and structural brain networks of tinnitus.

In summary, we applied pCASL MRI to delineate whole-brain CBF alterations in patients with tinnitus and, for the first time, explored sex-specific differences. Our results offer new insights into the neuropathological mechanisms underlying tinnitus and provide candidate imaging biomarkers that may inform future developments in precision diagnostics and individualized therapeutic interventions.

## Data Availability

The raw data supporting the conclusions of this article will be made available by the authors, without undue reservation.
